# Letter to the editor: Applying incidence-based disability-adjusted life years (DALYs) disease burden estimates to foster change in national vaccination policy, Slovenia, 2017 to 2018

**DOI:** 10.2807/1560-7917.ES.2018.23.27.1800354

**Published:** 2018-07-05

**Authors:** Mario Fafangel, Marta Grgič Vitek, Irena Klavs

**Affiliations:** 1 National Institute of Public Health (NIJZ), Ljubljana, Slovenia

**Keywords:** burden of illness, tick-borne encephalitis (TBE), vaccination, prioritisation in public health, public health policy

**To the editor:** Recently, Cassini et al. presented in *Eurosurveillance* the results of the burden of infectious diseases in European Union and European Economic Area (EU/EEA) countries [1], using the incidence-based methodology to calculate disability-adjusted life years (DALYs) developed within the Burden of Communicable Diseases in Europe (BCoDE) project [2]. The description of the impact of diseases on the health of the population by means of a composite health measure provides clear and comprehensive information for transparent and accountable decision-making. Thus, measures such as DALYs have the potential to play a significant role in health policy formulation.

We wish to add our first-hand experience using the BCoDE incidence-based approach to foster change in national vaccination policies. In a study published in 2017, based on the same methodology, we estimated the burden of tick-borne encephalitis (TBE) in Slovenia [3]. Compared with other EU/EEA countries, notification rate of TBE in Slovenia is among the highest, ranging from eight per 100,000 population in 2009 to 15 per 100,000 population in 2013 [4]. In terms of burden, we estimated that TBE in Slovenia during 2009–2013 accounted annually for 11.0 DALYs per 100,000 population (95% uncertainty interval (UI): 10.2–11.7), much higher than the pooled results published by Cassini et al. for selected EU countries (including Slovenia) in the same time period, which was 0.69 DALYs per 100,000 population (95% UI: 0.65–0.74) [1,3]

Vaccination against TBE has to be paid out of pocket for the majority of the population (except for occupationally exposed persons), with overall coverage remaining low at 7% [5]. With limited resources hindering the ability to offer universal and free TBE vaccination to the general population, evidence was needed for targeting vaccination to groups where the disease impact is highest, in order to yield the greatest benefit for the health of the population. Incidence of TBE in Slovenia was highest in the 50–74 years age group; however, we identified the highest burden of TBE among children aged 5–14 years. We concluded that incidence alone did not fully reflect the impact of disease and advised to complement epidemiological information with burden of disease estimates to inform vaccination policy.

Based on our results, a proposal for TBE vaccination policy change was on the agenda at our National Immunisation Technical Advisory Group (NITAG) on 15 June 2016 [6]. Our results, in addition to other factors such as routine surveillance data, specifics of the national immunisation programme linked to implementation of vaccination and expert opinions of specialists at the National Institute of Public Health (NIJZ) were considered by NITAG. Together they led NITAG to advise the introduction of publicly reimbursed TBE vaccination for children at 3 years of age and for adults at 45–50 years of age. The respective proposal prepared by NIJZ was considered by the Health Council at the Ministry of Health and approved on 10 May 2017 [7]. At the time of writing, negotiations are ongoing to ensure funding to implement the decision in 2019.

In our experience, the use of the methodology and toolkit developed by Cassini et al. provided a comprehensive view of the burden of disease on population health and helped us identify priority groups for intervention [8]. The disability-related impact of TBE on different age groups would not have been detected if we had limited our assessment to incidence alone, thus ignoring the combined effects of morbidity, short- and long-term sequelae and mortality, which are taken into account in the proposed BCoDE methodology. In our opinion, such an approach could benefit other EU/EEA countries by providing evidence-based information that allows for national and international comparisons, effective priority ranking of communicable diseases for public health action, identification of risk groups and a straightforward indicator to communicate public health needs to policymakers.
